# Supplementary material for: 76-year decline and recovery of aspen mediated by contrasting fire regimes: Long-unburned, infrequent and frequent mixed-severity wildfire
Source: PLoS One. 2021 Feb 4;16(2):e0232995. doi: 10.1371/journal.pone.0232995 (PMC7861421; doi:10.1371/journal.pone.0232995)
Supplement: S1 Table — (DOCX) [file pone.0232995.s001.docx]

S1 Table. Study stand attributes.

| Stand | Forest | FF | FS | Site | Stand area (ha) 1941 | Stand area (ha) 1993 | Stand area (ha) 2017 | Edge:area ratio 1941 | Edge:area ratio 1993 | Edge:area ratio 2017 | % conifer 1941 | % conifer 1993 | % conifer 2017 | # polygons 1941 | # polygons 1993 | # polygons 2017 |
| --- | --- | --- | --- | --- | --- | --- | --- | --- | --- | --- | --- | --- | --- | --- | --- | --- |
| 1 | Lass | 2 | LM | both | 3.07 | 0.32 | 0.70 | 0.09 | 0.25 | 0.22 | 35 | 52 | 12 | 5 | 8 | 12 |
| 2 | Lass | 2 | H | both | 1.61 | 0.03 | 0.23 | 0.07 | 0.35 | 0.35 | 42 | 78 | 5 | 3 | 2 | 10 |
| 3 | Lass | 1 | LM | both | 3.11 | 0.34 | 1.08 | 0.10 | 0.22 | 0.13 | 22 | 85 | 68 | 7 | 5 | 11 |
| 4 | Lass | 1 | LM | both | 0.84 | 0.00 | 0.02 | 0.11 | 0.00 | 0.51 | 8 | 100 | 62 | 2 | 0 | 1 |
| 5 | Lass | 1 | H | upl | 1.12 | 0.04 | 0.27 | 0.06 | 0.38 | 0.11 | 27 | 65 | 2 | 1 | 2 | 1 |
| 6 | Lass | 2 | LM | both | 1.92 | 0.00 | 0.22 | 0.07 | 0.00 | 0.19 | 15 | 100 | 10 | 3 | 0 | 3 |
| 7 | Lass | 0 | U | both | 0.22 | 0.10 | 0.07 | 0.15 | 0.28 | 0.34 | 10 | 57 | 67 | 3 | 3 | 4 |
| 8 | Lass | 0 | U | rip | 1.33 | 0.26 | 0.18 | 0.09 | 0.22 | 0.29 | 38 | 55 | 42 | 2 | 2 | 4 |
| 9 | Lass | 0 | U | both | 1.08 | 0.22 | 0.11 | 0.11 | 0.12 | 0.41 | 40 | 58 | 70 | 3 | 1 | 9 |
| 10 | Lass | 0 | U | both | 0.56 | 0.15 | 0.07 | 0.13 | 0.24 | 0.28 | 42 | 60 | 55 | 3 | 3 | 2 |
| 11 | Mod | 2 | LM | upl | 0.87 | 0.04 | 0.10 | 0.07 | 0.64 | 0.41 | 10 | 22 | 18 | 3 | 9 | 9 |
| 12 | Mod | 1 | LM | upl | 0.48 | 0.04 | 0.02 | 0.07 | 0.31 | 0.58 | 3 | 12 | 9 | 1 | 1 | 2 |
| 13 | Mod | 2 | LM | upl | 1.20 | 0.52 | 0.92 | 0.05 | 0.05 | 0.05 | 15 | 33 | 12 | 1 | 1 | 1 |
| 14 | Mod | 1 | LM | both | 1.30 | 0.65 | 1.50 | 0.06 | 0.08 | 0.06 | 12 | 20 | 15 | 2 | 1 | 1 |
| 15 | Mod | 1 | LM | upl | 4.48 | 1.12 | 2.54 | 0.04 | 0.10 | 0.07 | 3 | 12 | 8 | 2 | 2 | 3 |
| 16 | Mod | 0 | U | upl | 9.64 | 6.67 | 5.21 | 0.03 | 0.04 | 0.05 | 11 | 20 | 38 | 3 | 4 | 4 |
| 17 | Mod | 1 | LM | upl | 2.09 | 1.16 | 1.72 | 0.05 | 0.07 | 0.04 | 13 | 10 | 13 | 1 | 1 | 1 |
| 18 | Mod | 1 | LM | upl | 3.96 | 1.36 | 2.67 | 0.04 | 0.08 | 0.04 | 5 | 31 | 6 | 1 | 2 | 1 |
| 19 | Mod | 0 | U | upl | 1.20 | 0.00 | 0.00 | 0.04 | 0.00 | 0.00 | 2 | 5 | 7 | 1 | 0 | 0 |
| 20 | Plum | 2 | H | rip | 1.09 | 0.10 | 0.11 | 0.06 | 0.26 | 0.33 | 12 | 63 | 3 | 1 | 1 | 3 |
| 21 | Plum | 2 | H | rip | 0.63 | 0.06 | 0.19 | 0.09 | 0.23 | 0.12 | 32 | 70 | 0 | 2 | 1 | 1 |
| 22 | Plum | 2 | H | rip | 0.58 | 0.14 | 0.30 | 0.13 | 0.26 | 0.23 | 47 | 68 | 7 | 3 | 4 | 5 |
| 23 | Plum | 1 | LM | rip | 0.10 | 0.04 | 0.09 | 0.20 | 0.23 | 0.16 | 28 | 38 | 10 | 1 | 1 | 1 |
| 24 | Plum | 2 | LM | rip | 0.45 | 0.22 | 0.41 | 0.19 | 0.24 | 0.20 | 30 | 86 | 34 | 4 | 3 | 4 |
| 25 | Plum | 1 | H | rip | 0.68 | 0.15 | 0.91 | 0.11 | 0.25 | 0.12 | 47 | 79 | 0 | 3 | 2 | 5 |
| 26 | Plum | 1 | H | rip | 0.26 | 0.00 | 0.12 | 0.12 | 0.00 | 0.28 | 31 | 100 | 15 | 2 | 0 | 3 |
| 27 | Plum | 2 | H | both | 7.72 | 1.52 | 8.84 | 0.03 | 0.16 | 0.07 | 65 | 38 | 16 | 2 | 10 | 7 |
| 28 | Plum | 1 | H | upl | 0.33 | 0.08 | 1.11 | 0.08 | 0.24 | 0.10 | 62 | 78 | 8 | 1 | 1 | 2 |
| 29 | Plum | 1 | H | upl | 1.18 | 0.06 | 0.31 | 0.05 | 0.28 | 0.18 | 15 | 70 | 0 | 2 | 2 | 2 |
| 30 | Plum | 1 | LM | both | 0.08 | 0.05 | 0.22 | 0.16 | 0.24 | 0.21 | 59 | 64 | 32 | 1 | 1 | 2 |
| 31 | Plum | 0 | U | both | 8.69 | 3.43 | 2.58 | 0.04 | 0.07 | 0.08 | 11 | 26 | 37 | 1 | 3 | 4 |
| 32 | Plum | 0 | U | upl | 9.23 | 1.43 | 1.22 | 0.02 | 0.07 | 0.06 | 7 | 58 | 58 | 1 | 2 | 2 |
| 33 | Plum | 0 | U | both | 11.11 | 4.05 | 3.93 | 0.04 | 0.05 | 0.05 | 20 | 48 | 52 | 6 | 4 | 3 |
